# Effectiveness of Physical Therapy in Treating a Patient With Cervical Spondylosis Caused by Rheumatoid Arthritis to Improve the Quality of Life

**DOI:** 10.7759/cureus.62620

**Published:** 2024-06-18

**Authors:** Nikita Bhusari, Mitushi Deshmukh

**Affiliations:** 1 Musculoskeletal Physiotherapy, Ravi Nair Physiotherapy College, Datta Meghe Institute of Medical Sciences, Wardha, IND

**Keywords:** cervical spine, cervical spondylosis, hydrotherapy, rehabilitation, physiotherapy, rheumatoid arthritis

## Abstract

Rheumatoid arthritis (RA) is a disease characterized by symmetrical erosive polyarthritis that results in progressive disability. In patients with RA, the clinical course can be intermittent or progressive, depending on the severity of the symptoms. As complications of RA may develop within months of presentation, it is recommended that patients seek treatment with disease-modifying antirheumatic drugs as soon as possible. Wrists, proximal interphalangeal joints, metacarpophalangeal joints, and metatarsophalangeal joints are the most commonly involved joints, with the spinal joints and distal interphalangeal joints usually spared. The term cervical spondylosis refers to the general wear and tear of the cervical spine (neck) caused by aging, which can result in neck pain, neck stiffness, etc. Cervical degenerative spondylosis is caused by arthritic changes to the osseocartilaginous components of the cervical spine. This may result in compression of spinal nerve roots or the spinal cord, leading to pain in the neck, radiculopathy, or myelopathy. The present case report is of a 57-year-old female having pain in her neck and lower back for 20 years. She also complained of pain in the bilateral wrist joint, proximal interphalangeal joints, distal interphalangeal joints, metacarpophalangeal joints, and metatarsophalangeal joints. The pain was insidious in onset and gradually progressive. The pain has progressed to such an extent that it was hindering her day-to-day activities. The pain progressed on movement and was relieved with rest and medications. The pain was not associated with tingling or numbness. Physical therapy aims to strengthen weak structures, lessen pain, increase joint range of motion, improve movement patterns, increase cardiovascular endurance, and enhance the patient's quality of life.

## Introduction

A chronic inflammatory and degenerative joint condition, rheumatoid arthritis (RA) affects 0.5-1% of people in industrialized nations. It often leads to severe impairment and a decline in the quality of life. It is the most common rheumatic disease, with approximately 90% of patients experiencing some degree of wrist and hand pain [1]. An inflammatory disease with a wide range of multisystem comorbidities, RA is characterized by the uncontrolled proliferation of synovial tissue [2]. Typical examination findings include swelling, bogginess, tenderness, warmth, as well as atrophy of muscles surrounding the involved joints [2]. Women are more likely to suffer from this condition, with a 3:1 ratio. The most significant environmental risk factors are smoking, exposure to silica, and textile dust [3].

The following particular warning signs were identified in RA patients: acute exacerbation or increased complaints; unexplained persistent severe pain and inflammatory signs in one or more joints; recent tendon rupture (e.g. extensor digitorum, extensor pollicis, or biceps brachii muscle); symptoms related to the central nervous system (neck pain, in combination with paraesthesias and/or dysaesthesias, motor deficits, jumpy legs and/or a grainy sensation in the hands, incontinence, and tremors); peripheral neurological symptoms (sensory deficits, whether or not in combination with motor deficits, in the upper extremities, motor deficits, sensory, or circulatory deficits in the lower extremities) [4]. In order to diagnose, a variety of imaging techniques are available: plain radiography, Doppler ultrasound, and magnetic resonance imaging. The clinical course of most patients is progressive, and structural damage develops within the first two years. Pain relief and prevention of joint damage and functional loss are the major objectives of RA management. Disease-modifying antirheumatic medications (DMARDs) and non-steroidal anti-inflammatory medicines (NSAIDs) are used to treat RA. In the case of RA, the treatment for it should be undertaken with optimism, but such treatment can take a long time, and it differs greatly from patient to patient. The aim is to improve muscle strength as well as joint flexibility [5].

Neck pain is often referred to as "nonspecific (simple) neck pain," with symptoms related to postural or mechanical factors [6]. Cervical spondylosis is a progressive degenerative disorder of the human spine that is often associated with the natural aging process. Due to the osteophytic formations that occur as a result of progressive spinal segment degeneration, it is referred to as vertebral osteophytosis secondary to degenerative disc disease [7]. The patient may initially appear rigid and inflexible at the head and neck due to growing axial neck pain with cervical spine movement. The cervical paraspinal muscles, the periscapular muscles of the neck, and/or the superior trapezius muscles frequently have sensitive "trigger" points [8].

In most cases, there is no need for further investigation, and the diagnosis is made solely based on clinical evidence. Plain radiographs of the cervical spine may reveal a loss of normal cervical lordosis, indicating muscle spasm. However, most other signs of degenerative disease are found in asymptomatic individuals and do not correlate with clinical symptoms. When more serious pathology is suspected in the cervical spine, magnetic resonance imaging is the investigation of choice [6]. The symptoms of cervical spondylosis include neck pain, cervical radiculopathy, and cervical myelopathy. Neck pain and cervical radiculopathy (nerve root involvement) can be acute, subacute, or chronic conditions resulting from a variety of stages in the degenerative process. Surgical intervention should be reserved for patients who have failed conservative treatment, and whose symptoms cannot be adequately controlled by nonoperative treatments [9]. Approximately 25% of individuals with cervical spine spondylotic changes on radiographic imaging remain asymptomatic, whereas 50% of those aged over 40 and 85% of those aged over 60 exhibit signs of degeneration. C6-C7 are the most commonly affected levels, followed by C5-C6. Asymptomatic cervical spondylosis is generally characterized by neck pain [8].

In several studies, physiotherapy interventions, such as electrical stimulation, exercise programs, and manual therapy, have been demonstrated to reduce pain levels in individuals with RA. As a result of these interventions, overall function can be improved, thereby alleviating pain and improving quality of life. The use of physiotherapy is an essential part of comprehensive treatment for individuals suffering from RA. Physical therapy interventions can enhance outcomes and improve the quality of life for patients with RA by addressing physical impairments and functional limitations. RA patient’s quality of life is an important aspect of their care and management. A patient's perception of their physical, psychological, and social health is included in this measure. The measurement of health-related quality of life (HRQoL) may be useful for a number of reasons. HRQoL is more important to patients than disease-related variables, such as inflammatory biomarkers or joint counts. Assessments based primarily on narrow disease activity may not adequately describe the patient's primary health outcomes.

## Case presentation

The present case report is of a 57-year-old female having pain in her neck and lower back for 20 years. She also complained of pain in the bilateral wrist joint, proximal interphalangeal joints, distal interphalangeal joints, metacarpophalangeal joints, and metatarsophalangeal joints. The pain was insidious in onset and gradually progressive. The pain has progressed to such an extent that it was hindering her day-to-day activities. The pain progressed on movement and was relieved with rest and medications. The patient was prescribed methotrexate (15 mg once weekly), a disease-modifying antirheumatic drug, along with folic acid to reduce the risk of side effects, and nonsteroidal anti-inflammatory drug ibuprofen (400 mg) to reduce joint inflammation and pain. The pain was not associated with tingling or numbness. The patient also complains of pain in the bilateral knee for 15 years. She complains of difficulty in standing from a sitting position.

Clinical findings

**Figure 1:**
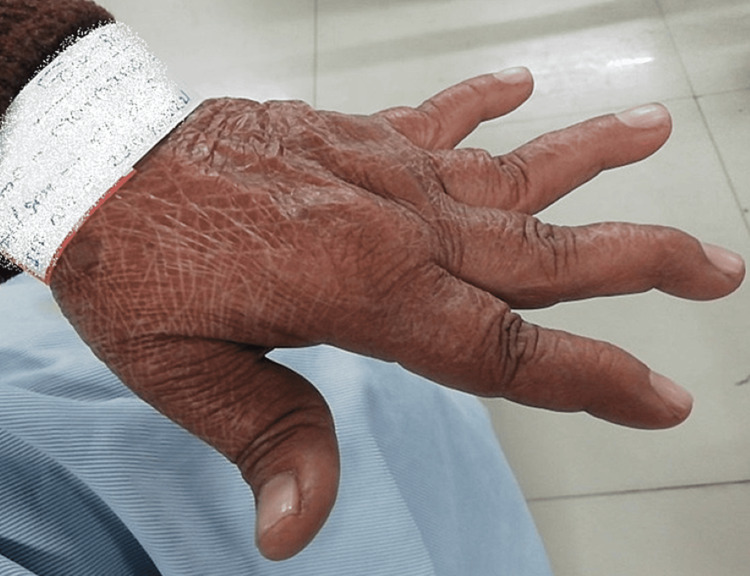
Deformity present in the interphalangeal joint

**Figure 2:**
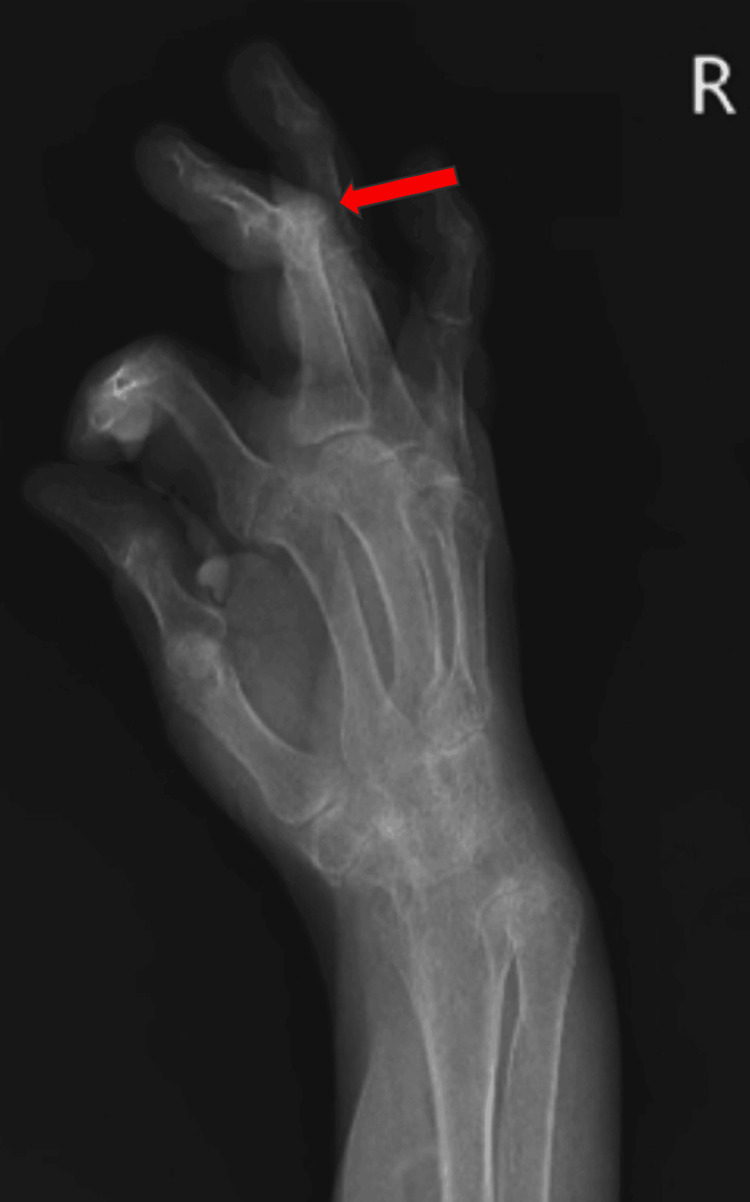
Radiograph showing the interphalangeal joint

Upon overall assessment, the vital signs remained steady. The patient appeared to be conscious and aware of his surroundings. The form of the body was ectomorphic. The numeric pain rating scale (NPRS) score was an 8. Palpation revealed grade 1 tenderness on the proximal and distal interphalangeal joints of both hands. Crepitus was present. The deformity was present in the interphalangeal joint as shown in Figure 1. As seen in Figure 2, an X-ray verified the joint displacement and reduced joint space. Blood investigations revealed elevated ESR and positive rheumatoid factor.

**Figure 3:**
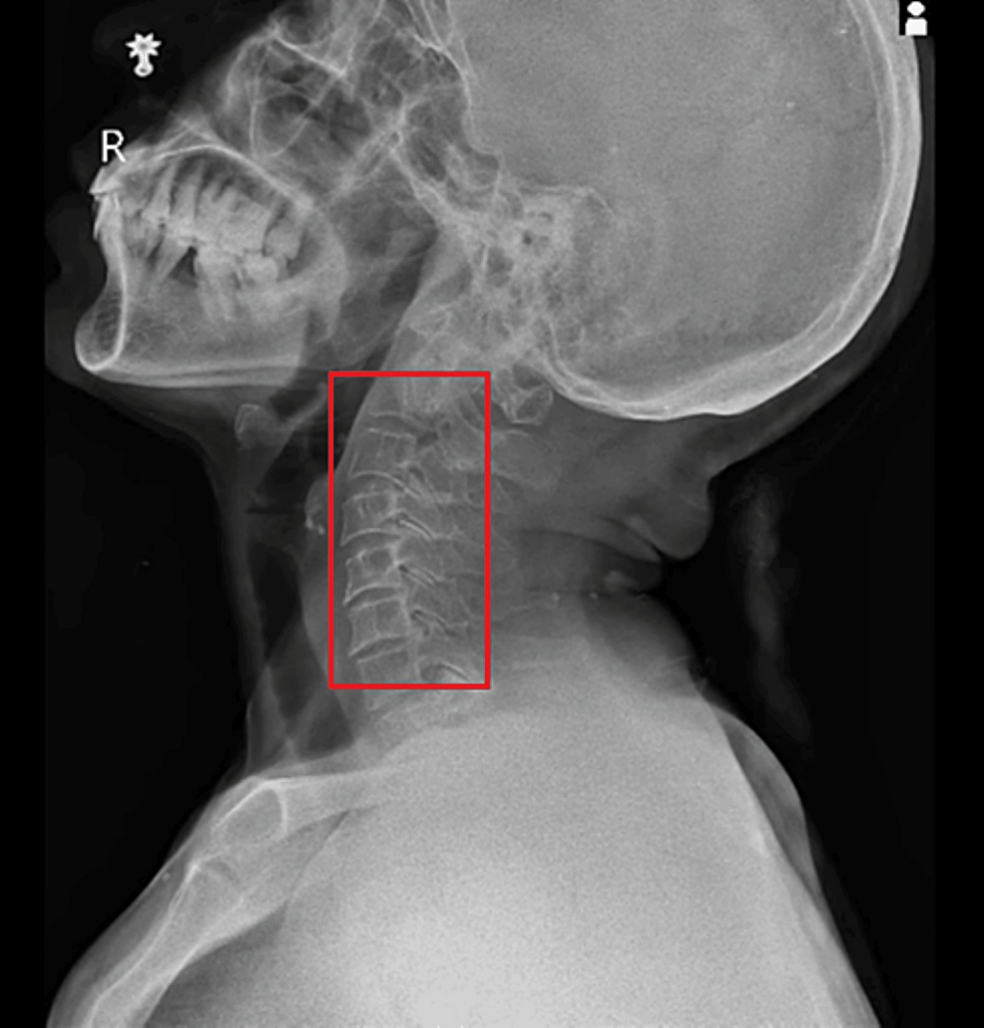
X-ray of cervical spondylosis

Figure 3 illustrates an X-ray of cervical spondylosis.

In this case study, assessment such as range of motion and manual muscle testing was taken, which are given in Tables 1-2, respectively.

**Table 1:** Range of motion

Joint	Joint movement	Pre-range of motion		Post-range of motion	
Hip		Left	Right	Left	Right
	Flexors	0°-80°	0°-70°	0°-90°	0°-77°
	Extensors	0°-20°	0°-20°	0°-23°	0°-25°
	Flexors	0°-70°	0°-65°	0°-75°	0°-70°
Wrist	Extensors	0°-55°	0°-45°	0°-60°	0°-50°
	Radial deviators	0°-10°	0°-15°	0°-15°	0°-15°
	Ulnar deviators	0°-20°	0°-20°	0°-25°	0°-25°
Cervical	Flexors	0°-40°		0°-50°	
	Extensors	0°-50°		0°-60°	

**Table 2:** Manual muscle testing

Joint	Joint movement	Pre-muscle strength		Post-muscle strength	
Hip		Left	Right	Left	Right
	Flexors	3/5	3/5	5/5	5/5
	Extensors	3/5	3/5	4/5	4/5
	Flexors	3/5	3/5	5/5	5/5
Wrist	Extensors	3/5	3/5	4/5	4/5
	Radial deviators	2/5	2/5	3/5	4/5
	Ulnar deviators	3/5	3/5	4/5	4/5
Cervical	Flexors	3/5		4/5	
	Extensors	3/5		4/5	

Physiotherapy management

Immediately following the patient's evaluation, physiotherapy intervention was initiated. The purpose of rehabilitation is to manage the consequences of disease. An initial assessment consists of a history taking, physical examination, and analysis. It is feasible to obtain a thorough grasp of the patient's health status during a physical examination and history taking. A comprehensive evaluation involves looking for warning signs and focal points in this summary. The analysis enables the prioritization of the patient's limitations and impairments, leading to the formulation of a treatment plan and goals. The goal of physiotherapy treatment is to help patients with RA manage their disease. A physiotherapist provides patients with joint protection strategies, assistive devices, and therapeutic exercises in conjunction with occupational therapists.

Cold/hot applications

The most common physical agents used in the treatment of arthritis are cold and hot modalities. Cold application is commonly used in acute stages of RA, whereas hot application is used in chronic stages. Heat is used to provide analgesia, to relieve muscle spasms, and to increase the elasticity of periarticular structures. The use of heat prior to exercise is beneficial for maximum results. There are various types of thermotherapy such as superficial hot packs, infrared radiation, paraffin, fluidotherapy, or hydrotherapy. A 10-20 minute application is recommended once or twice a day. A patient with sensory deficits and impaired vascular circulation in the hands and feet should be treated with caution. A cold application is preferred for active joints, where an increase in intraarticular heat would be undesirable.

The effects of heat on joints have been studied in several ways. Application of surface heat raises the intra-articular temperature [6]. The joint temperature dropped within the first five minutes, but then it naturally started to climb. It has been proposed that during the first few minutes, circulation shifts away from the inflammatory synovial tissue, and superficial arteries dilate. In contrast, cold treatments have the opposite result. Patients with inflamed joints experience different effects from typical, healthy ones when heat is applied.

Hydrotherapy

Although both concepts are now frequently used interchangeably, the term "balneotherapy" was originally intended to distinguish thermal and mineral water therapy from hydrotherapy. Balneotherapy has been used as a treatment option in different rheumatic disorders in recent years, especially in chronic degenerative diseases. Increased range of motion, strengthened muscles, relief from excruciating muscle spasms, and enhanced patient well-being are the goals of balneotherapy [10].

Electrical stimulation (ES)

The use of ES is used to relieve the pain experienced by patients with RA. Transcutaneous electrical nerve stimulation (TENS) therapy is the most commonly used therapy. Numerous studies have shown that using TENS once a week for three weeks reduced pain and increased hand grip strength after 15 minutes of use each day [7]. ES helps persons who are unable to actively recruit muscles to help with strength and endurance training [11]. Only a single, modest, high-quality study demonstrates how ES improves hand fatigue resistance and grip strength [4].

Outcome measures

The interpretation of outcomes such as the NPRS, rheumatoid arthritis disease activity index (RADAI), and rheumatoid arthritis quality of life (RAQoL) are given in Table 3.

**Table 3:** Outcome measures used to assess the effectiveness of intervention

Outcome measures	Pre	Post
Numerical pain rating scale	8/10	5/10
Rheumatoid arthritis disease activity index (RADAI)	7/10	3/10
Rheumatoid arthritis quality of life (RAQoL)	22/30	15/30

## Discussion

RA is a chronic systemic inflammatory disease that affects a significant percentage of the population. It is common for this disease to affect the cervical spine. Cervical spine involvement may also occur incidentally in asymptomatic patients. It is difficult to interpret the clinical manifestations of cervical disease in patients, with RA due to their joint arthropathy, muscle wasting, decreased range of motion, compression neuropathy, and poor functional status. Combining a range of motion exercises with strength exercises can improve grip and pinch strength, reduce pain, and maintain hand function more effectively than either range of motion or wax therapy alone [4]. In cases of severe joint inflammation, resting splints may be required at first. To prevent flexion contractures, patients with significant inflammation should engage in a daily passive full range of motion exercises [8]. As a general and nonspecific term, cervical spondylosis encompasses a wide range of conditions; however, it can be divided into three clinical syndromes for clarification: Type I (cervical radiculopathy); Type II (cervical myelopathy); and Type III (axial joint pain) [12]. In several trials and systematic reviews, physical therapy has been shown to be effective in treating cervical spondylosis and its symptoms [13]. Spondylosis derives from the Greek word spóndylos, which means vertebra. This term is used to describe wear and tear that affects the cervical spine over time, including the intervertebral disk, facet joints, and other connective tissues (e.g. cervical spinal ligaments) [14]. As spondylosis progresses, the disc undergoes degenerative changes where desiccation occurs, resulting in a reduction in disc height and the disc's ability to maintain or bear additional axial loads. Providers of organized work rehabilitation programs include occupational therapists and physiotherapists with training in ergonomics and work rehabilitation. In the treatment of cervical disk disease, physical therapists play an important role.

A typical treatment regime requires 15-20 sessions of 30 to 45 minutes each over a period of three months. In addition to supervised isometric exercises, proprioceptive re-education, and manual therapy, the treatment should be customized to each individual patient [13]. Informing and educating the patient on the intended course of treatment is crucial to the effective management of this illness. Preventing disability, increasing functional capacity, relieving pain, and educating patients are the main goals of physiotherapy and rehabilitation applications for RA patients [7]. RA patients' everyday living disabilities are much reduced, and their condition is considerably better managed with the help of physiotherapy and rehabilitation programs [7]. There is no doubt that physical therapy is of utmost importance at all stages of the disease and represents the most effective means of preventing chronic disability [6]. Physical therapy aims to strengthen weak structures, decrease discomfort, increase joint range of motion, improve movement patterns, boost cardiovascular endurance, and enhance patients' quality of life [8]. Joint exercises should be initiated as soon as painless motion can be achieved.

As part of this study, cold/hot applications, hydrotherapy, electrical stimulation, and balneotherapy were used as interventions. Following the intervention, the patients were evaluated with the outcome measures to determine whether the treatment had been effective. Pre- and post-intervention scores were obtained for outcomes such as NPRS, RADAI, and RAQoL; this shows a positive response to the interventions used.

## Conclusions

Although there is strong evidence that rehabilitation can enhance psychological status and function, little is known about its long-term benefits, who is most likely to benefit from it, or how effective it is in preventing or maintaining function when it is administered early in life. In addition to reducing pain and stiffness in the joints, physical therapy can also prevent deformities, aid in the restoration of function and improve quality of life and independence. Behavioral approaches to patient education, joint protection and energy conservation training, dynamic exercise therapy, hydrotherapy, and self-efficacy training are some of the interventions used to increase function, reduce discomfort, and improve physical and psychological status. Supportive footwear and orthoses are additional management strategies. Cognitive-behavioral therapy is one psychological technique utilized with individuals who have a lower psychological status. For patients with active or severe RA, intensive coordinated in-patient or day-patient rehabilitation is preferred. In this case study, a structured rehabilitation program is designed for the patient, with the goal of preparing them for both pre- and post-surgery recovery. Based on the outcome measures used in this study, the effectiveness of physical therapy can be explained.
